# Barriers, Facilitators, and Strategies to Improve Participation of a Couple-Based Intervention to Address Women’s Antiretroviral Therapy Adherence in KwaZulu-Natal, South Africa

**DOI:** 10.1007/s12529-023-10160-7

**Published:** 2023-02-28

**Authors:** Jennifer M. Belus, Lindani I. Msimango, Alastair van Heerden, Jessica F. Magidson, Valerie D. Bradley, Yvonne Mdakane, Heidi van Rooyen, Ruanne V. Barnabas

**Affiliations:** 1 Department of Medicine, Swiss Tropical and Public Health Institute, Basel, Switzerland; 2 University of Basel, Basel, Switzerland; 3 Department of Psychology, University of Maryland, College Park, MD 20742 USA; 4 Center for Community Based Research, Human Sciences Research Council, Pietermaritzburg, South Africa; 5 MRC/WITS Developmental Pathways for Health Research Unit, Department of Paediatrics, Faculty of Health Science, University of the Witwatersrand, Johannesburg, South Africa; 6 The Impact Centre, Human Sciences Research Council, Durban, South Africa; 7 Global Health and Medicine, University of Washington, Seattle, WA USA; 8 Present Address: Department of Clinical Research, University Hospital Basel, Basel, Switzerland; 9 Present Address: Division of Infectious Diseases, Massachusetts General Hospital and Harvard Medical School, Boston, MA USA

**Keywords:** Couple-based intervention, HIV, Medication adherence, Relationship discord, Disclosure

## Abstract

**Background:**

Couple-based interventions (CBIs), despite strong efficacy in improving numerous HIV risk behaviors, are not widely available and have not been tested to improve women’s antiretroviral therapy (ART) adherence. We examined barriers and facilitators to participation in a CBI based on cognitive behavioral couple therapy for women’s ART adherence in KwaZulu-Natal, South Africa.

**Methods:**

Semi-structured interviews were conducted with women with HIV (*n* = 15) and men of mixed HIV status (*n* = 15). Thematic analyses were guided by the Consolidated Framework for Implementation Research.

**Results:**

Facilitators mostly related to the couple’s relationship, including having an existing healthy relationship, men’s desire to support their partners, and a potential opportunity for men’s HIV disclosure. Barriers included a lack of understanding of how a CBI approach would be useful for women’s ART adherence, sole focus on women if male partners were also living with HIV, and men’s lack of prior HIV status disclosure to female partners.

**Conclusion:**

Findings indicate that relationship context and the male partner’s HIV status need to be addressed during recruitment, enrolment, and during the intervention to promote uptake.

**Supplementary Information:**

The online version contains supplementary material available at 10.1007/s12529-023-10160-7.

## Introduction

HIV-related illness and death continue to be among the leading causes of morbidity and mortality among women in South Africa, particularly those aged 15–24 [1]. South Africa currently has an estimated 7.8 million people living with HIV, of which over 60% are women [2]. Among women with HIV, approximately 58% are virally suppressed [3]. However, more needs to be done to support women’s consistent adherence to antiretroviral therapy (ART) in order to achieve the UNAIDS 95–95-95 goals and end the HIV epidemic [4]. This is particularly relevant in light of the fact that the majority of people with HIV will at some point in their lifetime experience challenges in the HIV care cascade [5].

Couple-based interventions (CBIs) represent a potential resource, albeit untapped, to support women’s ART adherence. CBIs are interventions that include partners in treatment, alongside the patient, and have successfully been used to improve several HIV-related behaviors. For example, Crepaz and colleagues [6] showed in a meta-analysis that CBIs (as opposed to individual interventions) resulted in increased odds (odds ratios between 1.51 to 1.79) of HIV testing, condom use, and nevirapine uptake to reduce mother to child transmission of HIV. Similar findings have been shown by other researchers [7, 8], including studies conducted in South Africa [9, 10]. To date, however, only two studies in the USA have tested the use of a CBI to improve ART adherence, and both found it to be superior to usual care in serodiscordant couples [11, 12].

There are several reasons to believe that a CBI would be appropriate for addressing women’s ART adherence in South Africa. First, many women with HIV are partnered or enter into new relationships after relationship dissolution [13], so working with women and their partners is realistic. Second, most women are in heterosexual relationships, in which men hold much power in the relationship, including over healthcare behaviors and decisions [14]. Third, prior qualitative work in South Africa supports that partners play a role in ART (non)adherence [15, 16]. Finally, a CBI targeting women’s needs may also be a better strategy to engage men in care. There is evidence that men, a challenging population to engage in HIV care [17], may be more receptive to participate in healthcare if it is through their female partner or for the betterment of their family [18–20].

Given this background, we sought to develop a CBI that had the following goals: improve women’s ART adherence, test whether men’s own engagement in care could be improved by virtue of their participation in a CBI, and improve the couple’s relationship functioning [21]. We therefore sought feedback on the following issues to inform intervention development: (1) the general idea of using a CBI to address women’s ART adherence, (2) specific intervention components and intervention structure that aligned with cognitive behavioral couple therapy (CBCT), the intervention’s theoretical framework, and (3) how a CBI focused on women’s ART adherence could affect men’s willingness to engage in the intervention and their own care as well as possible strategies to optimize this messaging. This study was conducted in accordance with the COREQ guidelines (see Appendix 1).

## Methods

### Participants and Procedures

Women and men (individuals, not couples) were recruited in April and May 2019 from Vulindlela, a semi-rural catchment area outside of Pietermaritzburg, in KwaZulu-Natal, South Africa. Study eligibility criteria for both women and men were as follows: aged 18 and over, in a committed heterosexual and monogamous romantic relationship (i.e., not in a polygamous relationship), resided in one of the neighboring communities where the study took place, and was conversant in IsiZulu (the dominant local language) or English. Women participants were also required to be living with HIV and self-report having difficulty with ART adherence. Self-reported ART adherence difficulty was assessed using two questions from the Ira Wilson assessment: number of days missed at least one dose of ART in the past 30 days and score on a 0 to 100 range of how often they take their ART the way they are supposed to [22]. Less-than-perfect responses to either question were considered non-adherent. There was no minimum time since HIV diagnosis or the couple’s relationship length that was required. Individuals who had previously participated in a CBI related to HIV prevention or treatment were not eligible to participate. Eligible individuals completed semi-structured interviews on the topic of barriers and facilitators to participation in the proposed intervention (focus of the current manuscript) and discussions of general healthy relationship functioning in the community [23].

Women were purposively recruited face-to-face using study contact cards, which were given to clinic staff at local clinics to identify women with HIV who had previously missed an HIV-related clinic appointment and/or were non-adherent to their ART. However, women’s self-reported HIV status and difficulties with ART adherence were used to determine study eligibility as we did not collect information from medical charts. Men were randomly approached at various community locations within the Vulindlela area (e.g., taxi rank) and screened for eligibility. A total of 15 women and 15 men contacted study staff or were approached, and all were eligible. Written informed consent was obtained by trained research assistants from all participants prior to conducting the individual interviews. Participants were paid ZAR 120 (~ 8.33 USD) for study participation. The study was approved by the local institution’s research ethics committee (#REC 3/19/09/18).

Two masters-level, bilingual research assistants from South Africa (LM, YM) received training and supervision on the semi-structured interview guide before conducting the interviews. Both research assistants had prior experience conducting qualitative interviews, were from the local community, and were gender-matched with participants. Interviews took place at the research site or in participants’ homes, depending on participant preference, and lasted approximately 60 to 90 min. Participants were not familiar with the researchers prior to study participation, nor did the interviewers share information regarding their personal interest in the research topic. All interviews were audio-recorded, translated into English, and transcribed by research assistants. Transcribed interviews were not shared with participants nor was their feedback sought on the findings. Field notes were written in English after each interview was complete.

Women participants were on average 38.6 (*SD* = 7.8) years old, 20% (*n* = 3) had a high school education or above, 20% (*n* = 3) were married or cohabitating with their partner, and approximately 87% had disclosed their HIV status to their partner (*n* = 13). Men who participated in the study were on average slightly younger at 32.4 (*SD* = 9.3) years old and had greater educational attainment, with approximately 73% (*n* = 11) having at least a high school education. Only two men were cohabitating or married to their partner. Regarding HIV status, *n* = 4 men were living with HIV, all of whom reported disclosing their status to their partner. The remaining men (*n* = 11) reported being HIV-negative, and approximately 73% had shared their HIV status with their partner.

### Proposed Intervention and Interview Guide

The semi-structured interview guide focused on the general idea of a CBI for women’s ART adherence and described core components of CBCT [24] in detail, which was the primary theoretical framework of the proposed intervention. Our intervention was framed as a “disorder-specific” CBI, based on the definition of Baucom and colleagues [25], which means that the intervention tries to improve both the patient’s well-being and the couple’s relationship. In this type of CBI, both partners are equally involved in the intervention, even if the intervention is targeted to improve only one partner’s health, in this case women’s ART adherence. This general idea was explained to participants at the outset of the interview. Moreover, a primary question of the pilot study (where the proposed intervention would be tested) was how framing a CBI for women’s health could potentially be used to engage men in care. During the interview, we sought feedback to optimize this framing.

With regard to intervention components, there are two primary skills taught in CBCT: communication skills and couple-level problem-solving skills. Communication skills involve teaching guidelines for the roles of speaker and listener in a conversation. This helps couples slow down their communication and facilitates understanding between partners. Couple-level problem-solving skills require the couple to jointly agree on a problem statement, share why the problem is important, brainstorm possible solutions, and implement a chosen solution on a trial basis [24]. Couples were provided with handouts of communication and joint problem-solving skill guidelines in isiZulu during the interview. The complete interview guide can be found in Appendix 2.

### Data Analytic Plan

The study employed thematic analysis [26], a qualitative data method and analytic approach used to identify relevant themes related to the study aims. Data analysis was informed by the Consolidated Framework for Implementation Research (CFIR) [27] to guide the identification of relevant themes. CFIR is an implementation science framework developed to organize research on what interventions work and the settings or contexts in which they work. Although there are five major domains in CFIR (the intervention, inner setting, outer setting, characteristics of individuals, and implementation process), we focused specifically on characteristics of individuals and intervention characteristics, as these domains were most relevant to our research question. In CFIR, characteristics of individuals typically focuses on providers, but this was adapted to focus on individual participants or couples for the current study, as we did not interview providers.

Transcripts were first reviewed by multiple study team members to familiarize themselves with participant responses. The research team subsequently developed a codebook, which identified codes, definitions, and example quotes. The research team involved in the study was comprised of bachelors, masters, and PhD-level academics from South Africa and North America. The team was diverse in age and academic experience (ranging from students to senior academics), gender (both women and men), and racial and ethnic identity (Black African, White, Asian/Indian, and mixed race).

The study used primarily inductive coding to capture participants’ feedback related to the use of a CBI to address women’s ART non-adherence, intervention structure, and intervention techniques. However, deductive coding was used to understand participants’ views on men’s participation in the intervention despite not being the focus of treatment. Sub-codes were developed within these larger themes. The first five transcripts were reviewed and an initial codebook was created using open coding. Additional transcripts were reviewed and the codebook was further refined. We found we were able to combine many of our initial codes during the focused coding process, suggesting that the dominant themes were being captured. Each transcript was coded by two trained coders, and discrepancies were resolved via consensus. When consensus was not possible, the first author served as the tiebreaker. NVivo was used to organize the data and support data analysis [28]. The second author used the codes to formulate themes presented in the “Results” section, under guidance of the lead author. Code frequencies, the co-occurrence of multiple codes, and code clustering were examined in NVivo. Findings were also compared across gender.

The research team aimed to identify and bracket existing preconceptions throughout the data collection and analytic process. During the data collection process, the interviewers were trained to highlight the challenging aspects of a CBI, such as being emotionally vulnerable, when describing the intervention components to participants. This helped ensure participants would have a realistic understanding of the intervention and be able to more readily describe challenges or barriers to participation. Furthermore, during the analytic process, we acknowledged our team’s preconceptions that were discussed as part of bracketing, which included beliefs that participants would inherently be interested in participating in a CBI, that HIV disclosure would emerge as a barrier or challenge to intervention participation, and that participants would be wary of discussing relational issues with an interventionist present.

## Findings

Both women and men perceived the intervention’s focus on women with HIV and ART non-adherence to be an important topic affecting the community. Participants initially struggled to directly respond to the question of whether a CBI approach could work to address women’s ART non-adherence because it was an unfamiliar intervention method. Participants gained more clarity as greater detail of the intervention approach was provided, including specific examples about the intervention techniques used. The overall perception of the intervention was positive, and there were a total of six themes regarding barriers and facilitators to intervention uptake. Two themes fell within the characteristics of individual/couple domain and four within the intervention characteristics domain. Some themes were identified as barriers or facilitators only, whereas others were viewed as both barriers and facilitators.

The primary themes within the characteristics of individuals/couples domain focused on the motivation for participating in a CBI for ART adherence, which were primarily relational. Specifically, these themes were (1) motivation to maintain or develop healthy relationship behaviors and (2) men’s desire to support their female partners. The primary themes within the intervention characteristics domain focused on how a dyadic intervention could actually meet the needs of both participating partners (or be adapted as needed to address both partners’ needs) as well as how working with an interventionist in a dyadic context would be experienced. These themes were (3) understanding why a CBI is useful for women’s ART adherence; (4) potential opportunity and challenge for men’s HIV disclosure; (5) dealing with negative reactions from partners during the intervention; and (6) comfort discussing relational issues with an interventionist. We describe each theme in detail below. Furthermore, we outline the themes in Table 1 and propose strategies that can be used to address barriers and capitalize on facilitators to increase uptake of the proposed CBI.

**Table 1:** Themes and suggested strategies for how to incorporate facilitators and address barriers to improve intervention uptake

Theme	Description of theme or subtheme	Outcome for participation	Strategy to (further) improve participation or uptake
Motivation to maintain or develop healthy relationship behaviors	Couple has an existing healthy relationship	Facilitator, potential barrier for couples with relationship difficulties	Clarify in the process of identifying potentially interested couples that those with relationship difficulties can participate in the intervention and may benefit from the skills
	Desire for improvement in communication or other aspects of the relationship outside of ART adherence	Facilitator	–
Men’s desire to provide support for female partners	Men want to participate in the treatment because they want their female partner to improve ART adherence	Facilitator	–
Understanding why a CBI is useful for women’s ART adherence	Unsure why men are needed in the intervention for women’s ART adherence	Barrier	Clarify why/how a CBI is useful for women’s ART adherence and how one partner’s adherence can affect the couple. Clarify that men will play an active role throughout the intervention rather than being passive observers. This message can be communicated during the recruitment and screening process and further reinforced with psychoeducation early in the intervention
Potential opportunity and challenge for men’s HIV disclosure	Men with HIV who have not yet disclosed their HIV status but desire to do so	Facilitator	Clarify that the intervention, through the support of a trained couples interventionist, can help couples successfully navigate conversations that otherwise would be more difficult to have
	Men with HIV who have already disclosed their HIV status and struggle with ART adherence	Barrier	Interventionist can assess a male partner’s ART adherence needs during the couple’s initial introduction to the intervention and treatment goal setting. This can be incorporated into the treatment interventions as needed
Dealing with negative reactions from partners during the intervention	Men with HIV who have not yet disclosed their HIV status and fear that participation in the intervention may lead to unexpected disclosure	Barrier	Re-iterate that the goal of the intervention is to support women’s ART adherence and that men will not be asked to disclose their HIV status. The presence of a trained couples’ interventionist can help the couple navigate challenging conversations, should an unexpected disclosure arise during the intervention
Comfort discussing relational issues with an interventionist	Presence of an interventionist who will observe relationship challenges	Facilitator for most, barrier for some	Normalize that couples may experience relationship challenges in the context of the intervention and that the interventionist is trained to help couples deal with such issues. Couples can experience the in vivo benefits of how a trained interventionist can help them navigate relationship challenges

### Motivation to Maintain or Develop Healthy Relationship Behaviors

Women and men described how couples who were already functioning well in their relationship would be very interested to participate in the intervention. They described couples with good communication skills, who spent regular time together, and who already supported each other as being motivated and comfortable participating:

Yes, I can do it [join the intervention] if my partner is with me. Because, my partner and I are always reminding each other about things, even when we are watching TV, we remind each other that we have to take our treatment… my partner and I know our time to take the treatment, we support each other a lot. We do not miss our clinic appointments. (Participant 02, female, age 47)

Encouraging couples with relationship difficulties to participate in the intervention during study recruitment is an important step to prevent only high relationship functioning couples from participating.

Participants also described how their interest in participating in the intervention extended beyond ART adherence into other areas of their life, particularly related to improving communication:

I will know that when we have a problem, which has nothing to do with taking medication, we will be able to know what to do and how we should communicate. Even if you have a problem with someone else and it is not your partner anymore, the knowledge you got here you, you will implement it there as well. (Participant 06, male, age 33)

Here, this participant described using the communication skills to address other problem areas in the relationship. Moreover, the participant viewed the communication skills as being transferable to future relationships with a new partner who was not part of the intervention. The idea of taking the skills learned and applying it to other relationships was echoed by other participants, who saw this as an opportunity to improve communication with other family members, including their own children:

It will be ok, because it can even help us improve the way we communicate with our children. When the two of you as a couple have a good communication system, it can have a positive impact on the children, and other family members. Because if the two of you have bad communication, it becomes hard to even discipline your children. (Participant 03, female, age 45)

### Men’s Desire to Support Their Female Partners

Participants believed that an intervention designed to facilitate men’s support of women would be well-received because they viewed proving support as one of the main roles of a partner. “I think a lot of them [men] would be happy because the purpose of being in a relationship is to support each other, and the women would feel happy to see their partners supporting them on this…” (Participant 19, male, age 23). Moreover, participants felt that men wanted to see their female partners take their medications consistently and learning the skills to best support women to do this was a welcomed intervention.

Most women generally shared the same sentiment that the role of a partner was to provide support, including for ART adherence. That said, some women viewed their male partner’s participation in treatment as a symbol of his love or commitment. “If the partner loves the women, he will come [to the intervention]. If he does not, he won’t” (Participant 07, female, age 33). In other words, some women viewed men’s participation in a CBI as something that only men who are committed to the relationship over the long-term would agree to participate in.

### Understanding Why a CBI Is Useful for Women’s ART Adherence

One potential barrier to intervention participation, mostly raised by men, was related to men’s lack of interest if it was unclear to them why they were being asked to partake in the CBI. “It’s about her and taking medication, and there is nothing where they say we need you as a partner to be there and support your partner to take medication and all, you see” (Participant 20, male, age 31). As expressed by the participant, there is a need to clearly explain the role that men play, both generally in supporting women to take their ART and specifically the role they will play in the intervention itself. Helping both partners understand how women’s ART adherence is in fact a couple’s issue is necessary, for example, by explaining how non-adherence can negatively impact caregiver responsibilities if one partner gets sick or how the male partner is likely needed to generate feasible, sustainable solutions for the problem going forward. As described in Table 1, the rationale for including men in this intervention can be provided during recruitment and screening, as well as reinforced early during the invention’s psychoeducation component.

This was further echoed by another participant who stated, “The man might have a negative attitude [about participating] even if the woman tries to convince him about it and the man would maybe say ‘that’s yours, not mine’” (Participant 28, male, age 23). As the quote suggests, it is important from the beginning that men have a clear understanding of how women’s ART adherence is actually a couple’s issue. If unclear, it may set up an unhelpful dynamic where women are put in the position of trying to convince their male partners to join the program.

### Potential Opportunity and Challenge for Men’s HIV Disclosure

We inductively explored participants’ expectations about how men viewed the idea of participating in an intervention where they were not the focus of treatment per se. For men whose HIV status was not disclosed or was unknown, the intervention was viewed as an opportunity for men to feel comfortable disclosing their status, given the focus on communication and presence of a counselor. “I think it can help them [men] as well to disclose to their partners their statuses because they will learn something about the importance of communicating to their partner and how it is going to help” (Participant 20, male, age 31). Not being the focus of the intervention was believed to make men feel more comfortable participating and engaging in the intervention:

It’s because sometimes we feel shy or ashamed of our status, so when you know nothing will put you in the spotlight you feel relieved. So, I think that will make them feel comfortable because it will be like they are just there for participating only and nothing else. Some people are afraid of being in the spotlight. (Participant 27, male, age 42)

At the same time, for men who are known to be living with HIV (i.e., have disclosed their HIV status to their partner prior to the intervention), the intervention’s singular focus on women’s ART adherence was viewed as a potential barrier to men’s participation. Men felt that if they were also struggling with ART adherence, they would want their concerns to be addressed in session as well. “They [men] will not be happy about that [intervention focused on women only], especially if they are both having a problem with taking their medication. They will feel excluded and lose interest” (Participant 24, male, age 32). This highlights the importance of integrating men’s ART adherence into the intervention, if the male partner’s adherence is a known issue. The extent to which the male partner needs ART adherence support can be assessed by the interventionist during the couple’s introduction to the intervention and treatment goal setting. In this way, it does not require men to focus on their own HIV treatment engagement but provides the opportunity for those men who would like to receive this support.

### Dealing with Negative Reactions from Partners During the Intervention

Both women and men expressed that a potential challenge with a CBI is that sharing opinions or feelings in session could lead to relationship conflict:

I think that sometimes it [discussing thoughts and feelings in session] could turn out and backfire, it could lead to you fighting. He might think that you talk too much or that you are now exposing him. He might also express something to me that would upset me, which could lead us to leaving the counselor’s office not seeing eye to eye or not talking to each other. (Participant 07, female, age 33)

There was also concern that information only known to one partner could be inadvertently revealed or discovered during the intervention. The issue of concern was mostly around men who knew they were living with HIV but had chosen not to disclose to their partner:

I have told him my status, but he has not disclosed his to me. So, he might think that in the intervention there might be a point that would lead him to disclosing it. That might cause us to fight because I am only finding out there that he also has HIV. Why did he not tell me because he knew mine? (Participant 14, female, age 31)

Couples will be reminded during the recruitment and intervention phase that men are not required to know or disclose their HIV status during the intervention. During the psychoeducation phase of the intervention, it is important to communicate to the couple that the interventionist can help couples navigate such difficult topics and conversations, should they emerge during the intervention.

### Comfort Discussing Relational Issues with an Interventionist

Most participants held positive views about discussing issues with their partner in front of an interventionist. They believed that an interventionist could act as a mediator between partners and potentially mitigate a partner’s negative reaction to something disclosed during the session by facilitating communication and helping the partners understand each other more readily:

I like the fact that there will be someone like you [a counselor] to help the couples, because I don’t have to talk to my partner alone. If he does not understand what I am saying to him, then you can clarify on my behalf. (Participant 18, female, age 28)

On the other hand, a few participants believed that the couple should discuss relationship issues alone first, come to an agreement, and then share the conclusion with the interventionist:

I think the problem here is that you might say something that will not sit well with her [the partner] and then she responds in a different way and then you find that your communication clashes. I think it would be better to first talk about it before we tell you [the interventionist] and then come with a solution. (Participant 22, male, age 26)

Participants who shared this perspective wanted to mitigate exposing relationship conflict to the interventionist and felt it was more appropriate for the couple to deal with the issue first. More psychoeducation can be included in the early phases of the intervention to help individuals understand how a trained interventionist can be used to facilitate challenging conversations between partners. Moreover, this can be naturally reinforced throughout the intervention, as the couple has in vivo experiences of the way a trained interventionist can help them navigate relationship challenges.

## Discussion

 CBIs offer an advantageous approach over individual-based interventions in improving several HIV-related outcomes [6–10]. Our ultimate goal is to add to this body of knowledge by examining the effect of a CBI targeting women’s ART adherence in KwaZulu-Natal. In preparation for a small pilot study, we conducted qualitative work to understand how community members from the target population perceived this approach. We found an overall positive view of working with couples to address women’s ART adherence, despite participants’ initial difficulty (albeit common) in understanding what a CBI approach entailed. Intervention facilitators mostly related to relational factors (e.g., couple’s relationship quality at baseline), whereas barriers mostly related to a lack of information or clarity on the intervention itself (e.g., limited understanding of how a CBI could be used to address women’s adherence issues). The HIV status of the male partner and his own disclosure and difficulties with ART adherence were perceived as both potential barriers and facilitators. Overall, findings provide clear steps for mitigating intervention barriers and building on intervention facilitators to support intervention uptake in the community to pilot test the intervention.

The primary facilitators to intervention uptake focused on relational aspects of the couple. Couples who had a positive relationship at baseline and men’s desire to support their female partners were both viewed as reasons why couples would want to participate in the CBI. Community members reported a high level of interest in learning the communication and joint problem-solving skills that would be taught in the intervention, which they viewed as being applicable to issues outside of ART adherence. Communication skills, and joint problem-solving skills to a lesser extent, have been found to be highly desirable skills in prior CBI studies [29, 30]. In low resource settings, where general couple or family therapy is very scarce, delivering CBIs or family-based interventions to target HIV or other physical or mental health concerns can also be a strategy to provide needed relationship or family support [29, 31].

It was also clear from the findings that participants felt men wanted to be in the role of supporter and see their female partners improve their ART adherence. This is consistent with prior work in Malawi, Tanzania, and Zimbabwe, which shows that when given the opportunity, men attend healthcare appointments and engage more in activities to support their partner or children’s health [18, 32]. For many men, being a “provider” for the family is central to their identity. However, the definition of what it means to be a provider extends beyond economic provision and can include supporting the safety, health, and well-being of female partners and children [33]. By bringing men into care to support women, it may also be an opportunity to address men’s own HIV needs, which often go unaddressed; however, this has mostly been examined in the context of antenatal care [34, 35]. CBIs and other family-based approaches may be a strategy to engage men in care for their own health and well-being.

A few potential barriers emerged around the role of men with HIV—how their adherence needs would be addressed in the intervention, lack of men’s disclosure prior to the intervention, or an unexpected HIV status disclosure and potential relationship fallout during the CBI. Data on the HIV concordance rate for couples in KwaZulu-Natal suggest that approximately one-third of people living with HIV enter into a relationship with a partner who is known to be living with HIV [13]. Given this, it is likely that some couples will present to this intervention where the male partner is also living with HIV and potentially struggling with adherence. Issues related to serodiscordant HIV status did not emerge from the interviews as a prominent issue. Perhaps because of the intervention’s focus (women’s ART adherence), general concerns about safe sex behaviors did not emerge. That said, it would be important to include key information related to HIV transmission and risk reduction strategies for serodiscordant couples in the intervention’s psychoeducation [36].

Study findings need to be considered in light of the study design and limitations. First, the use of qualitative data is not generalizable but does provide in-depth views from individuals who share a similar profile to those in the target population, at least for women. However, the extent to which male participants in this study reflect the target population, namely, men who are partnered with women with HIV who are struggling with ART adherence, is unclear. This creates more uncertainty regarding the male participant perspectives. Future research should consider enrolling dyads and triangulating findings across both members of the couple. Moreover, we relied on verbal descriptions of the intervention components and handouts of the intervention materials rather than a video demonstration or in vivo experience. This may have led to a less accurate understanding on the side of the participants as to what the intervention would entail.

Furthermore, this study assessed only community members’ views on intervention uptake and did not address systemic barriers and facilitators, such as those operating at the healthcare level. We also did not include perspectives of healthcare providers who would likely be delivering the intervention in the future. Such factors and perspectives will play a role in making the intervention available in the future and should be explored in future research. Finally, participants were given the option to have the interview conducted in their home or at the research field site. The different locations may have led to differing comfort levels answering the interview questions. Conducting interviews in the home setting can sometimes lead to privacy and confidentiality concerns because dwellings often have few rooms and other household members may also be present. However, we generally find that participants who choose to have their interview conducted at home are more forthcoming, likely because they view their home as a private space, which is also a familiar setting to them. It is important to provide participants with the option to choose a space where they will be most comfortable to answer interview questions in an open manner.

Overall, this study shows that community members perceive women’s ART adherence as an important area of focus and the use of a CBI using communication and joint problem-solving skills based in CBCT, as an acceptable approach to address this issue. Community members saw value in the intervention’s skills beyond their application to ART adherence or HIV. Furthermore, the identified barriers are factors that can likely be mitigated during the recruitment, enrolment, and early intervention sessions with psychoeducation. Findings provide support to further develop the intervention and study procedures and test the proposed CBI in the community.

### Supplementary Information

Below is the link to the electronic supplementary material.

Supplementary file1 (PDF 663 KB)

Supplementary file2 (DOCX 15 KB)

## Data Availability

Data supporting this article can be accessed by contacting the first author.
