# Supplementary material for: Barriers, Facilitators, and Strategies to Improve Participation of a Couple-Based Intervention to Address Women’s Antiretroviral Therapy Adherence in KwaZulu-Natal, South Africa
Source: Int J Behav Med. 2023 Feb 28;31(1):75–84. doi: 10.1007/s12529-023-10160-7 (PMC10803380; doi:10.1007/s12529-023-10160-7)
Supplement: Supplementary file 2 — Supplementary file2 (DOCX 15 KB) [file 12529_2023_10160_MOESM2_ESM.docx]

Appendix 2

*Semi-structured Interview Guide Questions*

| 1. The purpose of our treatment is to help HIV-positive women who are having trouble maintaining adherence to their HIV medications. This treatment, called START Together, has HIV-positive women and their male partners participate in the program together. The couple comes to every session together and learns problem-solving skills and communication skills. The treatment takes place with the couple only and the interventionist (not in a group). The treatment is not couple therapy, but the skills the couple learns, like problem-solving and communication, could help them in other parts of their relationship.    1. What are your initial reactions to this treatment idea? 2. Why do you think women who are HIV-positive [men] in your community would be interested in this type of intervention?   a. What are the potential barriers to their participation?   1. Part of the intervention will teach couples communication skills. When we do this, we teach couples how to be in the role of “speaker” and “listener” in each conversation *[give participant list of “speaker” and “listener” guidelines].* To practice this, you and your partner would have a conversation about a real issue related to your [your partner’s] HIV medication adherence problem, following these guidelines. The interventionist would guide you both through it, stopping each of you along the way to give feedback on how you’re doing and help you communicate better.    1. How would you feel about participating in this type of exercise with your partner?   Probes: What would be some barriers to your participation? What would go well? What would be hard?   - 1. How do you think HIV-positive women [men] in your community would respond to this exercise? Why?   2. As part of learning this skill, we encourage partners to disclose more personal feelings/experiences with their partners. For example, you might tell your partner that you’re scared to have HIV or your partner telling you that he’s worried that you’ll get sick. What this be like for you (to disclose such personal feelings)?   Probe: What would it be like for your partner?  Probe: Is there anything we could do when working with couples to make this easier for them?   1. Part of the intervention will teach couples joint problem-solving skills, that is, how to solve issues as a couple. When we do this, we teach couples to use their communication skills to share what’s important to them about the issue and then they brainstorm possible solutions together *[give participant list of problem-solving guidelines].* To practice this, you and your partner would identify a real barrier that’s getting in the way of you [your partner’s] taking your HIV medications, following these guidelines. The interventionist would guide you both through it, stopping each of you along the way to give feedback on how you’re doing and help you communicate better.    1. How would you feel about participating in this type of exercise with your partner?   Probes: What would be some barriers to your participation? What would go well? What would be hard?   - 1. How do you think HIV-positive women [men] in your community would respond to this exercise? Why?  1. How long do you think this intervention should be? Why? 2. I’ve told you a lot about the intervention and its components. Imagine you are now participating in the intervention with your current partner. How do you think it would go?    1. Would your partner be willing to participate? Why or why not?    2. What would you hope to get out of this treatment for yourself? What about for your partner?    3. What would be the hardest part about participating in this treatment? 3. This treatment focuses on helping HIV-positive women, so the treatment won’t be focusing on the HIV status of the male partners. How would men in the community feel about participating in treatment to support their partners, but not being in the spotlight (in other words, the treatment is not focused on them)?    1. Do you think HIV-positive men who haven’t disclosed their status to their partners would participate? |
| --- |
